# AI-peer integrated feedback in second language writing classes: exploring students’ engagement and writing performance

**DOI:** 10.3389/fpsyg.2026.1871951

**Published:** 2026-09-21

**Authors:** Wen Liu

**Affiliations:** School of Foreign Languages, Guangdong University of Science and Technology, Dongguan, China

**Keywords:** AI-peer integrated feedback, engagement, L2 writing, SCT, writing performance

## Abstract

While a considerable body of research has focused on examining the efficacy of peer feedback or AI-generated feedback in L2 writing, limited research has investigated the effect of the combination of AI and peer feedback on students’ engagement and their writing performance. To address this gap, the present study adopted a mixed-methods design. A 10-week feedback intervention was implemented with 122 Chinese EFL students over a 12-week study period: the experimental group (*n* = 61) received AI-peer integrated feedback, whereas the control group (*n* = 61) engaged in conventional peer feedback. To assess their engagement and writing performance, all participants completed pre- and post-tests, five writing assignments, and engagement questionnaires. Additionally, 16 students from each group participated in semi-structured interviews and kept reflective journals, providing rich qualitative data on their engagement patterns. The results indicated that the experimental group exhibited significantly higher levels of behavioral, affective, and cognitive engagement than the control group. Moreover, they demonstrated greater improvements across four writing subscales, namely task achievement, coherence and cohesion, grammatical range and accuracy, and lexical resource, albeit with varying magnitudes of gain across these dimensions. These findings suggest that AI-peer integrated feedback represents a promising pedagogical approach for fostering L2 writing development, shedding light on the potential for human-AI collaboration in language education.

## Introduction

Large class sizes are a significant barrier to providing prompt and personalized feedback in EFL writing courses (Yu, 2021). Therefore, peer feedback has gained recognition as an effective instructional tool, whereby students evaluate their peers’ written output and offer constructive comments, serving as an alternative to teacher commentary (Li et al., 2020). In addition to easing teachers’ burden of offering timely feedback, peer feedback fosters students’ critical thinking (Winstone and Boud, 2022). Moreover, it encourages students to learn from one another (López-Pellisa et al., 2021) and enhances academic writing proficiency (Noroozi et al., 2023).

Nevertheless, peer assessment efficacy can be undermined in the absence of ongoing oversight of peer feedback quality. Due to limited language proficiency, some students deliver low-quality feedback, leading to students’ skepticism toward peer feedback and discouraging them from actively incorporating such input (Lee, 2017). It has been argued that student involvement in peer assessment may be fruitless without external guidance, and that further assistance is essential for enhancing the value of peer feedback (Latifi et al., 2023). To mitigate this limitation, researchers suggest monitoring student-generated feedback to improve its quality. Proposed solutions involve teachers’ providing feedback on students’ peer evaluations (Han and Xu, 2020), a practice in which students scrutinize and respond to peer feedback during the assessment. Although authoritative and comprehensive, teacher feedback is often time-consuming and struggles to cater to individual students’ learning needs.

To address these limitations inherent in conventional feedback practices, generative AI represents a promising alternative to traditional feedback frameworks (Li, 2023; Steiss et al., 2024). Such technologies enable hybrid feedback configurations and can function as a supportive complement to peer feedback. Moreover, when used alongside human input, AI-generated comments offer L2 writers timely, ongoing guidance and consistent reinforcement (Noroozi, 2023). Despite growing calls for integrating AI and peer feedback, most existing research has examined students’ engagement with peer feedback and AI-generated feedback in isolation from each other. Empirical studies investigating learner engagement within AI–peer feedback condition remain scarce. Among the limited investigations, Zhang and Hashemifardnia (2026) explored tertiary-level L2 learners’ engagement with AI-peer feedback primarily through open-ended surveys, basing their findings on qualitative evidence from a small sample. This approach inevitably limits in-depth exploration of learners’ multifaceted experiences, constrains the interpretation of nuanced engagement, and weakens the generalisability of their findings.

To develop a more comprehensive understanding of how AI–peer integrated feedback influences L2 students’ learning and writing performance, the present study employs a mixed-methods design. Drawing on multiple data sources, including engagement questionnaires, semi-structured interviews, reflective journals, and students’ initial and revised writing drafts, this study investigates Chinese EFL learners’ affective, behavioral, and cognitive engagement with AI-peer integrated feedback, as well as how such feedback shapes their writing performance. This research aims to extend current understanding of L2 feedback engagement and to offer actionable pedagogical guidance for instructors seeking to foster meaningful student engagement when implementing this hybrid feedback practice.

### Literature review

#### Theoretical foundation

This research draws primarily on Vygotsky’s Sociocultural Theory (SCT) (1978) to frame the investigation into the effects of AI-peer integrated feedback on EFL students’ argumentative writing. SCT posits that human cognitive development is fundamentally socially situated, arising from meaningful interactions within social learning environments (Vygotsky, 1978). Within this framework, three interconnected core constructs, Zone of Proximal Development (ZPD), mediation, and scaffolding. ZPD represents the gap between students’ independent writing ability and the higher level of writing performance they can attain with tailored external support, thus highlighting the necessity of appropriate assistance matched to learners’ developmental potential (Vygotsky, 1978). Mediation, defined as the cognitive reorganisation prompted by interactive exchanges, positions feedback whether from human peers or artificial intelligence as an essential mechanism that steers learners towards enhanced performance (Poehner and Lantolf, 2024).

AI-peer integrated feedback design implemented in the current study resonates with the notion of scaffolding, which entails provisional assistance that allows students to tackle challenges located within their ZPD (Er et al., 2025; Tang et al., 2026). In this study, the experimental group received sequentially layered scaffolding: first, AI-mediated support provided individualized feedback targeting key writing dimensions, then peer-driven social mediation enabled learners to elaborate on and negotiate revision suggestions. Prior empirical work has affirmed the relevance of SCT to L2 writing, showing that social dynamics and contextual elements substantially affect the quality of writing (e.g., Mao and Lee, 2022; Abdi Tabari et al., 2025). Recent research has started to probe AI’s capacity to provide scaffolding within ZPD, indicating that AI-facilitated collaborative settings can foster interaction and help learners reach advanced competency levels (Liu et al., 2024). Building upon these empirical insights, the present study extends the SCT framework by examining how AI-peer integrated feedback enhances EFL learners’ writing performance in argumentative writing.

### Peer feedback in L2 writing

Feedback is fundamental to L2 writing development, as it offers corrective input and bridges the gap between current and target performance (Fong and Schallert, 2023). However, surging enrollments and large class sizes prevent teachers from delivering timely, comprehensive feedback (Latifi et al., 2023), thereby undermining its pedagogical effectiveness. Against this backdrop, peer feedback has gained recognition as a viable substitute for conventional instructor-led feedback (Abo et al., 2025). Distinct from teacher feedback, peer feedback creates a collaborative learning environment in which students educate one another largely independently, without minimal direct teacher involvement (Boud et al., 1999). Empirical studies have consistently shown that peer feedback enhances student’s writing quality (Xu et al., 2023). When students give feedback to their peers, they develop a greater understanding of genre conventions, strengthen their writing abilities, and cultivate reflective and critical thinking skills (Yu et al., 2019). Furthermore, the advantages of peer feedback extend beyond mere grammar or vocabulary correction; it also builds subject-specific expertise and enables students to assess their own academic progress more accurately than through passive feedback reception alone (Valero Haro et al., 2024).

Despite the potential of peer feedback in the learning process, various obstacles impede students’ productive engagement in such activities (Latifi et al., 2021). A primary challenge is the need for adequate training. Less advanced students frequently struggle to provide useful feedback and find it challenging to combine encouraging comments with critical analysis and to frame negative observations in a tactful manner (Gupta et al., 2019). Additionally, EFL students frequently question the validity of peer input, as teacher evaluations, which are associated with authority, are generally perceived as more reliable (Cheng and Zhang, 2024). Consequently, learners may disregard peer suggestions, thereby diminishing the learning potential of the feedback process. Moreover, the act of giving and receiving feedback imposes considerable cognitive demands, requiring critical appraisal and the formulation of actionable solutions (Noroozi, 2023). To overcome these barriers, external support, particularly from teachers, represents a valuable means of improving the quality of peer feedback (Deneen and Hoo, 2023). Despite the observed success of teacher intervention in improving peer feedback quality, such practice remains time-consuming and labour-intensive, particularly in large classes.

### AI-feedback in L2 writing

Given their rapid response and scalability, AI-driven feedback is particularly well-suited to tackling challenges within large-scale learning contexts, where delivering timely, personalized feedback would otherwise prove difficult for instructors (Xu et al., 2023). Driven by advances in natural language processing, generative AI holds the potential to promote educational equity by enabling more students to receive instructional feedback (Warschauer et al., 2023) within L2 contexts. Tools such as ChatGPT leverage generative AI to provide responsive, adaptable feedback, exposing students to real-world language use and refining their writing fluency and precision (Kim et al., 2023).

Empirical research has validated the capacity of AI tools to enhance learners’ academic performance. As Su et al. (2023) demonstrated, ChatGPT facilitates the development of EFL learners’ argumentative writing. Beyond delivering comprehensive feedback on content construction, idea organisation, and writing refinement, the tool also strengthens students’ learning motivation and self-efficacy. Similarly, Meniado (2023) revealed that generative AI systems can foster language-oriented learning via personalized feedback, thereby advancing L2 learners’ writing fluency. Beyond supporting individual writing development, AI also serves as a valuable facilitator of peer feedback activities (Darvishi et al., 2022). It enables students to generate high-quality peer feedback (Han et al., 2022) and engages them in the iterative cycle of drafting, shaping, and revising evaluative remarks (Guo, 2024). Collectively, these studies underscore the potential of integrating AI-driven tools to support and optimize instructional practices within L2 writing pedagogy.

### Engagement

Engagement is broadly understood as a multidimensional construct characterized by sustained effort and active involvement (Fredricks et al., 2019), and is widely considered an essential prerequisite for successful L2 acquisition. The most influential theoretical model for understanding student engagement was originally proposed by Fredricks et al. (2004), who conceptualized it as a meta-construct comprising three interrelated dimensions: affective, behavioral, and cognitive engagement. In recent years, a growing body of research has turned its attention to how L2 learners engage with feedback in writing contexts. Building upon Fredricks et al.'s (2004) foundational framework, Ellis (2010) proposed a multidimensional model specifically designed to capture learner engagement with both oral and written corrective feedback. In this model, behavioral engagement refers to learners’ adoption of feedback, reflected in specific revision actions. Cognitive engagement involves learners’ ability to perceive and comprehend feedback as well as their use of metacognitive strategies to support its processing; and affective engagement encompasses learners’ feelings toward feedback, their beliefs about its usefulness, and their emotional reactions to it. Expanding on Ellis's (2010) framework, subsequent studies have considerably deepened and refined these engagement sub-dimensions (e.g., Cheng and Liu, 2022; Shen and Chong, 2023; Zhang and Hyland, 2022). For example, Park and Kim (2019) adopted depth of processing as an analytical tool to operationalize cognitive engagement with feedback. In their framework, superficial processing was manifested by learners’ detection of errors and initial attempts at self-correction, whereas deep processing was characterized by learners’ generation of multiple alternative self-corrections, indicating more elaborate cognitive engagement.

Based on the above framework, the present study defines student engagement as a multi-dimensional meta-construct consisting of three interconnected components.

- Affective engagement denotes students’ attitudes toward feedback, which are measured through their level of interest, perceived usefulness, and emotional reactions (Fan and Xu, 2020).
- Cognitive engagement encompasses the cognitive processes activated by attending to feedback. It involves both the depth of processing feedback (e.g., noticing and comprehending its content) and the strategic application of cognitive and metacognitive strategies to interpret feedback and inform subsequent revisions (Koltovskaia, 2020).
- Behavioral engagement covers the observable steps students take in response to feedback. These include revision operations and strategic behaviors aimed at addressing the feedback received and improving learning outcomes (Zheng and Yu, 2018).

### Engagement with writing feedback

Traditionally, researchers focused on examining the efficacy of feedback, including dimensions such as feedback scope (Rahimi, 2021), timing (Fu and Li, 2022), and delivery modes (Karim and Nassaji, 2020). However, studies have shown that writing progress cannot be achieved through feedback alone, regardless of its type. What ultimately determines developmental outcomes is the manner in which students engage with feedback (Zhang and Hyland, 2022). By reconceptualizing students as active feedback users rather than passive recipients, recent research has shifted toward examining learners’ feedback receptivity and agentic use to unlock the benefits of feedback (Panadero and Lipnevich, 2022). Consequently, scholarly interest has increasingly centred on feedback engagement among L2 writing students.

Several recent studies have examined how students engage with peer feedback. For example, Yu et al. (2019), drawing on multiple qualitative data sources, found that postgraduate students displayed superficial behavioral and cognitive engagement with peer feedback in academic writing tasks, and that affective engagement played a dynamic mediating role between the other two dimensions. Similarly, Fan and Xu (2020) showed that L2 college students’ level of engagement varied depending on the type of feedback: learners demonstrated deep engagement when they received form-focused peer feedback, yet only superficial engagement when they received content-focused feedback. Although these studies have generated valuable insights into L2 writing development, most have adopted case study approaches drawing primarily upon interview data and learners’ journals, with insufficient analysis of textual evidence from learners’ drafts. Consequently, these findings may be undermined by a lack of methodological triangulation, as interpretations depend largely on self-reports rather than on observable behavioral responses (Zhang and Hyland, 2022).

Another line of research has examined L2 learners’ engagement with AI feedback. Koltovskaia et al. (2024) found that among six Iranian graduate students, behavioral engagement centered on resolving lower-order writing issues, cognitive engagement on recognizing and critically evaluating AI feedback, and affective engagement on a positive disposition toward using ChatGPT for paraphrasing and stylistic refinement. Wang and Xue (2024) reported similar gains across all three engagement subdomains among Chinese undergraduate EFL learners using AI chatbots, emphasizing that such tools provide targeted scaffolding that enhances emotional, behavioral, and cognitive involvement in writing-related feedback activities. Despite these affordances, inconsistent empirical evidence has also surfaced regarding the implementation of AI feedback in pedagogical settings. Teng and Huang (2025) examined Chinese EFL undergraduates’ engagement with ChatGPT feedback through a pre-post survey on affective, behavioral, and cognitive engagement. Their results showed that ChatGPT significantly enhanced affective and behavioral engagement (e.g., higher motivation and participation), whereas cognitive engagement, though slightly improved, did not differ significantly between groups, suggesting limited impact on deeper critical thinking. Generally, these mixed findings underscore the complexity of embedding AI tools in educational practice and call for further inquiry into how ChatGPT-supported feedback affects learners’ engagement.

Beyond existing research focusing on separate feedback sources, limited studies have explored learners’ engagement with the combination of AI and peer feedback. To date, Zhang and Hashemifardnia (2026) have conducted one of the few empirical comparisons between AI-peer feedback and peer feedback. Their study targeted Iranian postgraduate English majors, examining the intervention’s influence on writing accuracy alongside three engagement dimensions: vigor, dedication, and absorption. All participants completed pre- and post-writing tests and an engagement scale, while the AI–peer feedback group completed open-ended questionnaires about AI-assisted feedback. The results demonstrated significant gains in writing accuracy and engagement within both conditions. Despite its contributions to understanding the effects of hybrid feedback on learners’ engagement, the study has clear methodological limitations. Its qualitative dataset was limited to open-ended survey responses. Without supplementary interviews or focus groups, the researchers could not thoroughly investigate participants’ nuanced experiences, which weakens the depth and trustworthiness of thematic analysis. Moreover, its modest sample size limits cross-context generalizability. Accordingly, further large-scale mixed-methods research is required to comprehensively evaluate the effectiveness of AI–peer integrated feedback across varied learning environments.

Previous research has investigated the effectiveness of peer feedback, AI feedback, and their combination on learners’ engagement, yet notable limitations persist: studies on peer feedback often lack methodological triangulation; research on AI feedback has yielded divergent findings regarding learner engagement; and existing AI-peer feedback investigations have relied on modest sample sizes without focus group or interview data. Against this backdrop, the present study examines learners’ behavioral, cognitive, and affective engagement with AI–peer integrated feedback among 122 Chinese EFL students. It employs methodological triangulation, drawing on both self-reported data (questionnaires, semi-structured interviews, reflective journals) alongside objective evidence from learners’ written revision behaviors to corroborate findings.

This research aims to determine whether AI–peer integrated feedback influences learners’ engagement and their writing performance. Specifically, this study addresses the following two research questions:

*RQ1*: To what extent does AI-peer integrated feedback facilitate EFL learners’ engagement with feedback?

*RQ2*: To what extent does AI-peer integrated feedback enhance EFL learners’ writing outcomes?

## Methods

### Participants

This study employed convenience sampling to recruit participants from three intact classes at a university in southern China. Rather than individual randomization, class-level allocation was adopted: two classes (A and B), each with approximately 30 students, were combined to form the experimental group (*n* = 61), while a single intact class (C) of 61 students served as the control group. All participants were English majors enrolled in a 12-week Advanced English Writing course, which consisted of two 45-min sessions per week. The same instructor who holds a master’s degree in applied linguistics with 4 years of teaching experience taught all the classes, following identical curriculum content and instructional methods. The participants have an average age of 20.2 years (SD = 0.77), with 98 female and 24 male students, reflecting the typical gender imbalance in Chinese English-major cohorts. Their overall English proficiency corresponded to Level B2 on the Common European Framework of Reference for Languages (CEFR), as indicated by their average College English Test Band 4 (CET-4) scores, a national English proficiency exam for university students in China (Jin et al., 2022).

Prior to the study, all participants were informed of the research purpose and potential benefits, and written informed consent was obtained from each participant. Strict confidentiality was maintained throughout the study, with participants assured that their responses and performance data would be used solely for academic research purposes and that their personal identities would remain anonymous.

### Instrument

#### Writing tests

This study employed pretest and posttest assessments to measure participants’ writing performance. The writing task was an argumentative essay, a widely used format for assessing L2 writing proficiency. The prompts, sourced from the IELTS Writing Task 2 item bank (see Appendix A), were selected for two primary reasons. First, empirical evidence has shown that argumentative writing poses significant challenges for many L2 learners (Alavi et al., 2020). Second, because IELTS Task 2 topics are grounded in everyday life experiences, they are equitable and accessible to all participating students. During each assessment, students were allocated 45 min of in-class time to complete a writing task of at least 200 words, with no access to external resources. The pretest and posttest were administered under identical conditions, including consistent writing genres and time limits.

#### Pre- and post-engagement questionnaires

To examine students’ engagement with AI-peer integrated feedback during the intervention, questionnaires were administered at Week 1 (pre-intervention) and Week 12 (post-intervention). Participants completed the electronic questionnaire during a 15-min in-class session and submitted their responses to the instructor. The questionnaire (see Appendix B) was developed based on Ellis’s (2010) three-dimensional engagement framework and drew on previous empirical studies on L2 writing feedback engagement (e.g., Cheng and Zhang, 2024; Zhang and Jiang, 2022). The questionnaire aimed to assess three core dimensions of engagement: affect, behavior, and cognition. Participants indicated their level of agreement with each statement using a 5-point Likert scale, where 1 represented “strongly disagree” and 5 represented “strongly agree.”

To assess the construct validity of the questionnaire, an exploratory factor analysis (EFA) was first conducted. The EFA yielded a three-factor solution consisting of 16 items, which accounted for 73.2% of the total variance (KMO = 0.86, *p* < 0.001). The three factors were labeled as affective engagement (5 items; e.g., “I enjoy engaging with the feedback I received”), behavioral engagement (5 items; e.g., “I carefully read and review all the feedback”), and cognitive engagement (6 items; e.g., “I can clearly comprehend the meaning and intent of the feedback”). Regarding internal consistency, the Cronbach’s α coefficients for the three subscales were 0.81, 0.77, and 0.79, respectively, all surpassing the acceptable threshold of 0.7. To rigorously validate the three-factor structure, a confirmatory factor analysis (CFA) was subsequently performed. CFA results confirmed the three-factor structure with good fit (χ^2^/df = 1.83, CFI = 0.96, TLI = 0.95, RMSEA = 0.059, SRMR = 0.045), and all items loaded significantly on their intended factors (loadings = 0.56–0.81). The AVE (0.52–0.58) and CR (0.78–0.81) values all exceeded the recommended benchmarks, supporting both convergent and discriminant validity.

#### Semi-structured interviews and reflective journals

Given that students’ engagement could not be fully captured through direct observation, self-report instruments such as interviews were considered necessary. Accordingly, 16 focal participants from each group took part in one-on-one semi-structured interviews (see Appendix C) designed to examine their cognitive, behavioral, and affective engagement with the respective feedback approaches. To help students feel more comfortable, the interviews were conducted in Chinese, each lasting approximately 30 min. Recognizing that the time limitations of semi-structured interviews might lead to incomplete data, participants were then asked to write reflective journals of approximately 150 words in either English or Chinese immediately after the interviews (see Appendix D). In these journals, participants documented their perceptions of the feedback they received, the revision strategies they employed, and the cognitive processes involved. To triangulate the interview and journal data, the study also collected the focal students’ initial drafts and their revised versions (i.e., first and second drafts across the five intervention writing tasks). These written materials helped identify patterns of feedback uptake and revision behaviors, offering a nuanced understanding of how L2 students engaged with AI–peer integrated feedback versus traditional peer feedback.

### Research design

This study adopted a quasi-experimental pretest-posttest design. The overall study spanned 12 weeks in total, while the feedback intervention lasted for 10 weeks. Specifically, in Week 1, all participants completed a pre-test that included both an argumentative essay to measure their baseline writing proficiency and an initial engagement questionnaire to assess their affective, cognitive, and behavioral engagement with feedback. Following the pre-test, the intervention was implemented from Week 2 to Week 11. During this period, participants took part in five writing sessions conducted at two-week intervals. In Week 12, participants completed the post-test and the second engagement questionnaire, while focal students took part in semi-structured interviews and completed reflective journals (see Figure 1).

**Figure 1:**
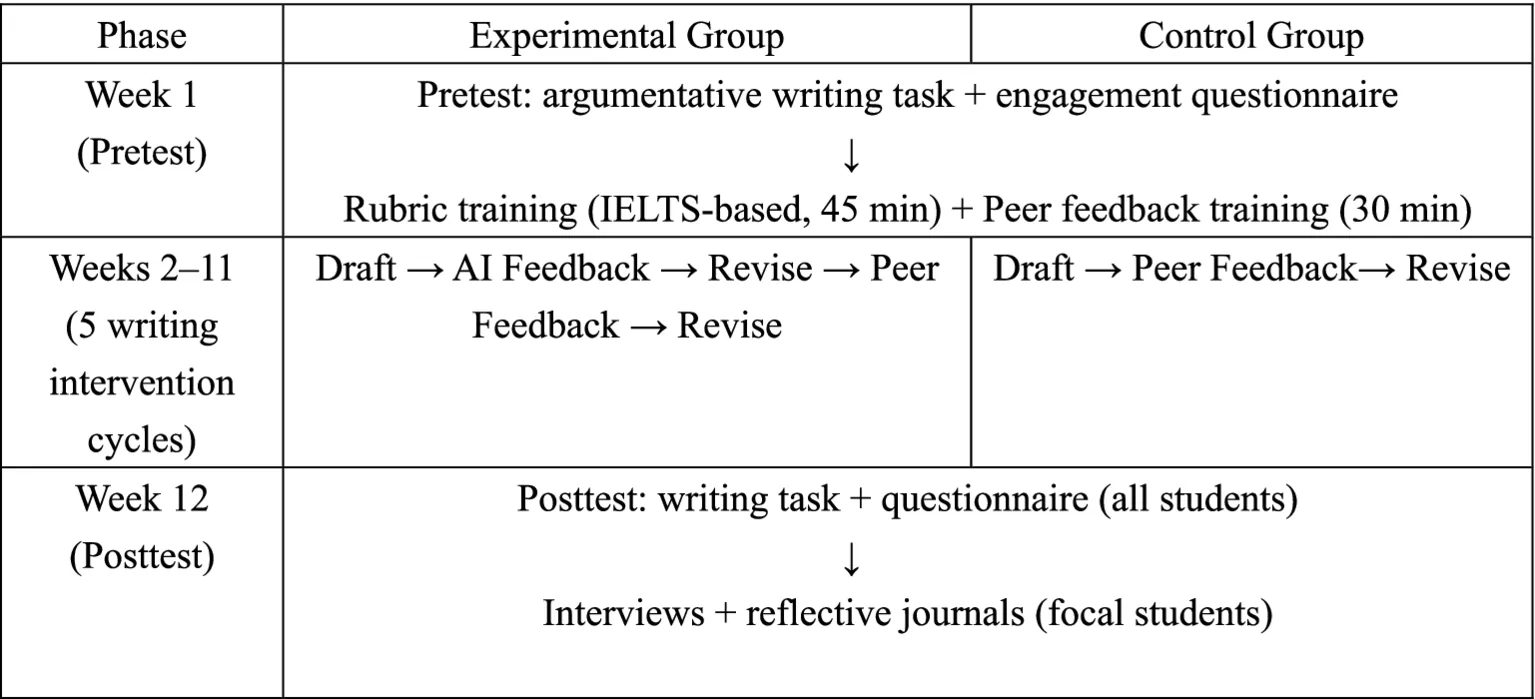
Research procedure.

#### Before intervention

After students completed the pretest and engagement questionnaire, the instructor delivered a 45-min training session on the analytic scoring rubric, ensuring that students understood the criteria defining effective L2 writing. The rubric employed in this study was adapted from the IELTS writing band descriptors, which centred on four key dimensions: task achievement, coherence and cohesion, lexical resource, and grammatical range and accuracy (University of Cambridge ESOL Examinations, 2011). Following the detailed explanation of each rubric component, students from both groups were led through self-assessment of their own written compositions according to the established criteria.

Furthermore, all participants participated in a 30-min peer feedback training session after completing writing task. During these training sessions, the instructor emphasized the importance of providing targeted, constructive feedback that addressed both local linguistic features and global rhetorical issues, along with the consistent and accurate application of the band descriptors. Throughout the intervention, the instructor assumed a facilitative and supportive role, overseeing instructional activities, resolving procedural queries, and monitoring adherence to the standardized rubric-based assessment framework.

#### During intervention phase

After completing the initial draft of each writing task, participants in the experimental group submitted their written work to ChatGPT-4 (GPT-4, web interface) for automated feedback. The model was run with its default parameters (temperature = 0.7, top_p = 1.0) to maintain a stable output style. All participants accessed the tool via individual free-tier accounts to ensure equal access privileges. For each draft revision cycle, students interacted with the AI tool for approximately 30 min, using a set of standardized prompts to receive targeted feedback on task achievement, coherence and cohesion, lexical resource, and grammatical range and accuracy. Students then revised their drafts based on the AI-generated suggestions. To ensure procedural consistency of AI-generated feedback across all participants, the instructional prompts were fully standardized. The instructor provided a structured template during preparatory training to regulate interactions with the AI platform. All participants were required to adopt the core prompt as the primary instruction, and any supplementary prompts were required to remain focused on the four pre-defined assessment dimensions to avoid divergent feedback outputs.

The core prompt, refined through pilot testing, was phrased as follows: “Provide constructive feedback on this essay focusing on task achievement, coherence and cohesion, lexical resource, and grammatical range and accuracy. Identify specific areas for improvement and suggest revisions, explaining the rationale behind each suggestion”. In addition, participants were allowed to use supplementary prompts to elicit detailed feedback, such as: “Identify instances of grammatical errors in this essay and suggest alternative phrasing” and “Evaluate the clarity and logical flow of the argument presented in this essay, providing suggestions to improve”. Although students were encouraged to make minor adaptations to these prompts to address their individual writing needs, they were instructed to remain aligned with the key dimensions of the assessment rubrics introduced in the earlier training. To promote responsible and effective use of the AI tool, the instructor periodically reviewed students’ interaction logs with ChatGPT, focusing not on the essay content itself but on the quality and appropriateness of their prompting strategies and their responses to AI feedback, in order to detect any inappropriate or ineffective prompting practices. Upon completing the AI-assisted revision, participants moved on to conventional peer feedback activities. Each peer review session consisted of 30 min of individual draft evaluation using the standardized rubric, followed by 15 min of small-group discussion (groups of 2–4 students) to exchange feedback and negotiate suggestions. Students then revised their essays based on the peer feedback they received. This cyclical instructional procedure was carried out biweekly across five successive writing tasks.

By contrast, participants in the control group engaged in peer review without AI support. After receiving their peers’ argumentative essay drafts, students spent approximately 60 min conducting independent evaluation and revision using the same standardized rubric. Similarly, they took part in 15 min of small-group discussion (groups of 2–4 students) to share comments and negotiate revision suggestions. Following the peer feedback phase, students revised their written compositions based exclusively on peer feedback.

#### Post-intervention stage

In Week 12, both groups completed a posttest and a second engagement questionnaire to measure gains in their writing performance and changes in engagement. At the end of the intervention, 16 focal students from each group also took part in semi-structured interviews and submitted reflective journals, yielding rich qualitative insights into how learners perceived and engaged with the respective feedback environments.

### Data analysis

#### Evaluation of students’ writing performance

In this study, two independent raters were recruited to score the writings from both groups. A total of 244 written compositions were collected and assessed using the IELTS Task 2 band descriptors on a 9-point scale. The overall score for each composition was calculated as the average of the four sub-component scores, with final results reported in 0.5-point increments across the 9-band scale. To ensure assessment reliability, 20% of the writing scripts were randomly selected and rated by both evaluators. The inter-rater reliability coefficients across the four dimensions were acceptable: task achievement (*r* = 0.86), coherence and cohesion (*r* = 0.81), lexical resource (*r* = 0.82), and grammatical range and accuracy (*r* = 0.80). Any discrepancies in ratings were later resolved through discussion between the two raters.

#### Analysis of semi-structured interviews and reflective journals

All interview audio recordings were transcribed verbatim, and the transcripts were cross-checked against the original audio files to ensure accuracy. The interview transcripts and students’ reflective journals were translated into English and then manually analyzed to explore how focal students engaged with the different feedback approaches used in the intervention. First, all textual data were read and reread repeatedly to gain familiarity with the qualitative dataset. Next, an inductive coding approach was employed (Creswell, 2014), following the sequential procedures of open, axial, and selective coding as outlined by Corbin and Strauss (2008).

Guided by the established engagement framework, the data were coded along three interrelated dimensions: affective, behavioral, and cognitive engagement. Corresponding subcodes were developed to capture nuanced responses, including students’ attitudes toward the value of different feedback modes, the revision strategies and operations they used when processing feedback, their awareness and comprehension of the feedback, and the cognitive and metacognitive operations they employed during revision. To identify and code relevant segments, the interview transcripts and reflective journals were carefully examined, with a focus on content related to students’ perceptions of and reactions to the distinct feedback interventions. After this coding process, cross-case comparisons were performed to synthesize and interpret the main findings.

To ensure the trustworthiness of the qualitative analysis, two independent coders separately coded approximately 20% of the dataset. Inter-coder reliability coefficients were 90.12% for affective engagement, 91.22% for behavioral engagement, and 91.06% for cognitive engagement. Any coding disagreements were resolved through in-depth consensus discussions between the two coders until full agreement was reached.

#### Analysis of feedback and students’ writings

A textual analysis was performed to characterize feedback practices and examine students’ behavioral engagement in detail. The feedback provided on students’ essays was analyzed at the level of individual feedback points, which were defined as any written interventions (Hyland, 2003). In this study, feedback points referred to the written commentary provided by AI and peers and were treated as meaningful units of analysis. Based on the engagement framework developed for this study, feedback was categorized according to its focus. Following the approach of Mao and Crosthwaite (2019), two types of feedback were distinguished by focus: global feedback and local feedback (see Table 1). By comparing students’ first drafts with their final revised versions, the study identified revision operations, which were subsequently classified into six categories according to the scheme proposed by Zhang and Hyland (2018): no correction, correction, addition, deletion, substitution, and reorganisation (see Table 2).

**Table 1:** Taxonomy of feedback focus.

Feedback focus	Definition	Illustrative example
Local feedback	Comments on grammatical or linguistic accuracy	Reading articles enrich our knowledge” (enriches)
Global feedback	Comments on the overall content and structural arrangement	This information does not support the main argument

**Table 2:** Classification of revision operations.

Category	Description
No correction	No changes made in response to feedback
Correction	Errors in grammar, mechanics, or sentence structure properly fixed
Addition	Provision of new words, phrases, sentences, or content
Deletion	Removal of existing words, phrases, sentences, or paragraphs
Substitution	Existing words or phrases are replaced with alternatives
Reorganization	The order of multiple sentences or paragraphs is rewrite or rearranged

To ensure the reliability of the coding process, two independent coders performed textual analysis on the students’ writing. Inter-coder agreement stood at 92.21% for feedback focus and 90.13% for revision operations, and any coding discrepancies were resolved through iterative discussion.

### Statistical analysis

A series of quantitative analyses were conducted to compare the outcomes of the experimental and control groups. For students’ engagement, three separate one-way ANCOVAs were performed on the posttest scores of behavioral, affective, and cognitive engagement, with the corresponding pretest scores entered as covariates. Given that three separate ANCOVAs were run, a Bonferroni-corrected alpha level was adopted (α = 0.017). For students’ writing performance, a 2 (group) × 4 (writing dimension) mixed-design ANCOVA was conducted, with participants’ overall pretest composite writing score entered as a covariate to control for baseline writing proficiency. The primary focus of this analysis was the group × writing dimension interaction, which tested whether the intervention’s effectiveness varied across the four writing criteria (task achievement, coherence & cohesion, lexical resource, and grammatical range & accuracy). All assumptions, including normality, homogeneity of variances, and sphericity, were verified. Greenhouse–Geisser correction was applied when sphericity was violated. To probe significant interactions, Bonferroni-adjusted comparisons were performed in two sequential steps: (a) simple effects to examine group differences within each dimension, and (b) pairwise comparisons among the four adjusted marginal means to determine whether the magnitudes of improvement differed across dimensions. Effect sizes (partial η^2^ and Cohen’s d) with 95% CIs are reported, and the significance level for the writing analysis was set at α = 0.05.

Given the quasi-experimental design (intact-class assignment), this study computed Intra-class Correlation Coefficients (ICCs) to assess potential data clustering. The ICCs were uniformly low across all outcome measures (affective engagement 0.041, behavioral engagement 0.033, cognitive engagement 0.047, task achievement 0.052, coherence & cohesion 0.038, lexical resource 0.044, grammatical range & accuracy 0.049). Most values fell below the conventional threshold of 0.05; only task achievement slightly exceeded this cutoff but remained very close to the criterion. Due to the limited number of classrooms available for this quasi-experiment, multilevel modelling was not statistically viable. These results indicate negligible within-class dependency, justifying the use of ANCOVA (for engagement) and mixed-design ANCOVA (for writing) as appropriate analytical frameworks.

## Results

### Quantitative analysis

#### Students engagement in both groups

Table 3 presents the descriptive statistics for the engagement questionnaires administered to both groups at the two time points. Independent-samples t-tests indicated that no significant differences between the two groups at the pretest stage in affective engagement (t(120) = 0.19, *p* = 0.847, d = 0.03), behavioral engagement (t(120) = 0.32, *p* = 0.749, d = 0.06), or cognitive engagement (t(120) = 0.21, *p* = 0.834, d = 0.04). These findings suggested that the two groups were comparable at the baseline level.

**Table 3:** Pre- and posttest descriptive statistics for engagement.

Dimension	Group	Pretest		Posttest	
		M	SD	M	SD
Affective engagement	Experimental	2.98	0.74	3.94	0.82
	Control	3.00	0.76	3.04	0.79
Behavioral engagement	Experimental	2.96	0.78	3.95	0.84
	Control	3.00	0.77	3.06	0.81
Cognitive engagement	Experimental	3.01	0.72	4.08	0.80
	Control	2.99	0.74	3.01	0.77

#### Affective engagement

ANCOVA revealed a significant main effect of group on posttest affective engagement after controlling for pretest scores (*F*(1, 119) = 49.32, *p* < 0.001, partial η^2^ = 0.29). The experimental group reported significantly higher adjusted mean scores (M = 4.03, SE = 0.09, 95% CI [3.85, 4.21]) than the control group (M = 3.05, SE = 0.09, 95% CI [2.87, 3.23]), mean difference = 0.98, 95% CI [0.70, 1.26]. This finding indicated that learners receiving the AI-peer integrated feedback condition displayed higher emotional involvement and perceived value of feedback activities relative to their counterparts in the control group.

#### Behavioral engagement

After adjusting for pretest scores, ANCOVA indicated a significant main effect of group on posttest behavioral engagement (*F*(1, 119) = 47.15, *p* < 0.001, partial η^2^ = 0.28). The experimental group demonstrated significantly higher adjusted mean scores (M = 3.94, SE = 0.09, 95% CI [3.76, 4.12]) compared to the control group (M = 3.07, SE = 0.09, 95% CI [2.89, 3.25]), mean difference = 0.87, 95% CI [0.59, 1.15]. This outcome showed that students in the experimental group tended to report more intensive behavioral investment, which aligned with qualitative observations regarding revision operations and strategies.

#### Cognitive engagement

ANCOVA for cognitive engagement also yielded a significant main effect of group (*F*(1, 119) = 57.03, *p* < 0.001, partial η^2^ = 0.32). The experimental group exhibited significantly higher adjusted mean scores (M = 4.07, SE = 0.09, 95% CI [3.89, 4.25]) than the control group (M = 3.02, SE = 0.09, 95% CI [2.84, 3.20]), mean difference = 1.05, 95% CI [0.77, 1.33]. These results suggested that students in the experimental group tended to devote greater cognitive resources to processing the AI-peer integrated feedback.

### Students’ writing performance in both groups

Table 4 presents the descriptive statistics for the four writing dimensions at pretest and posttest. Independent-samples t-tests revealed no significant baseline differences between the experimental and control groups across any dimension: task achievement (t(120) = 0.21, *p* = 0.836, d = 0.04), coherence and cohesion (t(120) = 0.23, *p* = 0.817, d = 0.04), lexical resource (t(120) = 0.22, *p* = 0.829, d = 0.04), and grammatical range and accuracy (t(120) = 0.20, *p* = 0.842, d = 0.04), confirming baseline equivalence prior to the instructional intervention.

**Table 4:** Descriptive statistics for both groups across the four writing dimensions at pretest and posttest.

Dimension	Group	Pretest		Posttest	
		Mean	SD	Mean	SD
Task achievement	Experimental group	16.82	2.45	21.48	2.18
	Control group	16.91	2.38	18.54	2.42
Coherence and cohesion	Experimental group	14.23	1.92	18.18	2.11
	Control group	14.31	2.05	15.82	2.23
Lexical resource	Experimental group	13.95	1.86	17.85	2.34
	Control group	14.02	1.79	15.28	2.19
Grammatical range and accuracy	Experimental group	15.47	2.51	19.71	2.06
	Control group	15.38	2.44	17.26	2.31

A 2 (group) × 4 (writing dimension) mixed repeated-measures ANCOVA was conducted, with the composite pretest writing score entered as a covariate. The analysis revealed a significant main effect of group (*F*(1, 119) = 42.68, *p* < 0.001, partial η^2^ = 0.26, 95% CI [0.14, 0.37]), indicating that the experimental group outperformed the control group after statistically adjusting for baseline writing proficiency. The main effect of writing dimension was also significant (*F*(2.85, 342.00) = 18.45, *p* < 0.001, partial η^2^ = 0.13, 95% CI [0.06, 0.21]). Most importantly, group × writing dimension interaction reached statistical significance (*F*(2.85, 342.00) = 2.93, *p* = 0.036, partial η^2^ = 0.024, 95% CI [0.001, 0.07]), justifying follow-up simple-effect analyses to probe whether intervention gains varied across the four writing dimensions.

Simple-effect analyses were subsequently performed to unpack this significant interaction. All four dimensions showed significant between-group differences favoring the experimental group, with effect sizes descending as follows: task achievement (d = 1.41, 95% CI [1.04, 1.77]), lexical resource (d = 1.28, 95% CI [0.92, 1.63]), coherence and cohesion (d ≈ 1.20, 95% CI [0.85, 1.55]), and grammatical range and accuracy (d = 1.16, 95% CI [0.81, 1.50]). Bonferroni-corrected pairwise comparisons on adjusted marginal change scores further revealed that the improvements in task achievement were significantly greater than gains in coherence and cohesion (*p* = 0.023). Likewise, lexical resource yielded significantly greater improvements relative to coherence and cohesion (*p* = 0.031). No other cross-dimension comparisons reached statistical significance (all *p* > 0.05). Taken together, AI–peer integrated feedback yielded significant gains across all four writing dimensions, though the pattern was differential. Task achievement and lexical resource improved most substantially, significantly outperforming coherence and cohesion. Meanwhile, coherence and cohesion showed the smallest yet still significant gains, and grammatical range and accuracy, while recording the lowest numerical increase, did not differ significantly from the other dimensions.

### Qualitative analysis

The qualitative findings derived from engagement questionnaires, semi-structured interviews and reflective journals were organized into three themes. These qualitative results converged with the quantitative patterns of students’ engagement and writing performance.

### Improved affective engagement with AI-peer integrated feedback

The data indicated that AI-supported feedback substantially enhanced students’ willingness to engage in iterative revision. Unlike traditional peer feedback, which often entails waiting for peer responses, the immediacy of AI-generated commentary appeared to encourage timely engagement with written work. One experimental group participant stated in the reflective journal, “Getting instant feedback made me want to revise my essay immediately. I didn’t have to wait for my teacher or classmates to check it”. This ready availability of feedback seemed to blur the boundaries between composing and revising, embedding revision as an ongoing component of the writing process rather than a discrete, delayed task. In addition to immediacy, the precision of AI feedback appeared to enhance motivation by providing clearer direction for revisions. For example, one student explained, “I never enjoyed revising before, but seeing AI identify exactly what needed fixing made me eager to correct everything and raise my score”. This comment suggests that specific guidance helped students perceive revision as valuable rather than routine. The straightforwardness of AI-generated feedback might have offered concrete targets, thereby strengthening students’ dedication to their writing. Unlike students in control group, who often worried about social evaluation (e.g., “they might think my writing is stupid”), participants receiving AI-supported feedback benefited from a non-judgmental environment that allowed them to identify and correct errors without interpersonal anxiety. One student from the experimental group noted in her reflective journal, “Knowing that I could check my mistakes anytime with AI made writing more interesting. I wasn’t scared of making errors because I knew I could fix them”. This theme signals that AI feedback mitigates affective barriers, enabling learners to take greater risks during drafting and revision.

### Active behavioral processing with AI-peer integrated feedback

The qualitative data revealed marked differences between the two groups in both revision operations and strategies. In terms of revision operations, the control group students predominantly confined themselves to direct correction or no correction, while the experimental group students exhibited a broader repertoire, encompassing correction, substitution, and addition. This wide range of operations suggested that AI-peer integrated feedback enabled learners to move beyond surface-level error fixing toward more substantive textual refinement. As one experimental-group student explained in the interview: “*The immediate and precise feedback I received from both AI and peer sources on linguistic and global dimensions enabled me to deploy a broader repertoire of revision operations, ranging from minor corrections to substantial rewriting of entire sentences*.” Distinct patterns also emerged concerning cognitive strategies for processing feedback. Under peer-only feedback, learners frequently adopted a receptive stance toward peer comments. Ambiguous or delayed peer input, together with interpersonal considerations, discouraged them from probing or challenging peer suggestions, and many accepted feedback without further critical inquiry. In the AI-peer integrated feedback condition, by contrast, participants enacted more agentive cognitive strategies when processing feedback. They proactively mobilized multiple external informational resources, including the AI tool, reference materials, and online sources, to interrogate and contextualize feedback input. They demonstrated critical utilization of these external supports. One participant described this iterative revision practice: “*This AI enabled me to revise the essay as many times as I wanted before finally submitting it. It was just like having a personal tutor helping me improve*. *I not only corrected the errors but also checked the AI’s suggestions against a grammar book to understand the rules*.” Likewise, another student documented in the reflective writing how she drew upon external resources: “*I liked how AI gave suggestions instead of marking something wrong. That made me think how I should fix this problem. When I received feedback on sentence structure, I looked up similar patterns online and practiced writing my own examples to remember them.”*

### Deep cognitive engagement with AI-peer integrated feedback

Concerning the awareness of feedback, students in both groups reported that noticing feedback and understanding most of the suggestions was not difficult. Nevertheless, a few participants from both groups acknowledged that some feedback was challenging to interpret. As one participant from the control group noted, “*I sometimes found it hard to understand peer comments on sentence structure, especially when the feedback was vague or lacked explanation*.” In contrast, several participants from the experimental group responded that sometimes they had difficulty understanding AI suggestions. As S5 pointed out, “*While AI systems were capable of identifying local writing errors, they sometimes failed to provide feedback on global-order concerns such as coherence and organization*.”

More importantly, the two groups exhibited divergent patterns in their deployment of cognitive and metacognitive strategies. Control-group students relying solely on peer feedback appeared to invest limited cognitive effort, often making only superficial, plausible changes without verifying accuracy or reflecting on underlying rules. This passive approach seemed attributable to the vagueness and delay inherent in peer feedback, which discouraged deeper engagement. In contrast, students in the experimental group used a range of cognitive and metacognitive strategies to monitor their mental efforts and handle AI-peer integrated feedback. Firstly, they actively analyzed and evaluated the feedback they received. Rather than accepting AI suggestions uncritically, they scrutinized their correctness and assessed whether the proposed changes aligned with their intended meaning. As one participant from the experimental group elaborated: “*AI feedback tended to be fallible, so I had to analyze and evaluate it to see whether it matched what I intended to express. If it did, I adopted it*.” Second, memorization emerged as a notable cognitive strategy. When encountering metalinguistic explanations of errors, some experimental-group participants deliberately committed these explanations to memory to deepen their understanding of error patterns and prevent recurrence in future writing. Similarly, one student reported taking notes to memorize synonyms and alternative expressions offered by AI, aiming to expand her lexical repertoire. Third, participants in the experimental group frequently connected feedback processing with prior knowledge. When facing revision challenges, they retrieved previously acquired instructional content to inform their revisions. One student’s reflective journal illustrated: “*After receiving AI feedback on underdeveloped ideas, I recalled an earlier teacher-led lesson on elaboration strategies, specifically, making comparisons and applied it to strengthen my argument*.” Fourth, planning emerged as a metacognitive strategy unique to the experimental group. Given the sequential nature of the AI–peer integrated feedback design (AI feedback first, followed by peer feedback), students strategically prioritized their revision efforts across phases. One participant stated in the interview: “*I planned to fix language errors right after getting AI feedback, and then I could spend more effort revising content and organization aspects highlighted by peers.”*

## Discussion

This study explored how EFL students engaged with AI-peer integrated feedback and how such engagement affected their writing performance across multiple dimensions. The findings indicated that participants demonstrated deep engagement with this feedback approach, which might in turn be associated with improvements in writing performance. These results indicated a link between student engagement and writing outcomes, providing empirical support for the view that engagement serves as a crucial mediator of feedback effectiveness (Cheng and Zhang, 2024). As previous research has suggested (e.g., Mao and Lee, 2022; Zhang and Hyland, 2022), simply providing feedback does not automatically lead to writing development. Rather, it is learners’ engagement with that feedback that drives improvement.

### Students’ engagement in both groups

With regard to affective engagement, both quantitative and qualitative data revealed that the experimental group, which received integrated AI-peer feedback, demonstrated significantly deeper affective engagement than the control group, which received peer feedback only. This finding corroborated Yuan (2025), who documented the affective benefits of AI-supported feedback for EFL writers. The capacity of AI to deliver rapid, constructive, and non-judgmental feedback might foster a more enjoyable and less stressful learning experience, strengthening students’ emotional connection to the revision process. In contrast, peer feedback alone often entails interpretive ambiguities, which could generate frustration. More importantly, AI-generated feedback is perceived as more supportive and personalized. Such a nature might effectively bolster learners’ self-efficacy. According to Jia et al. (2024), elevated self-efficacy predicted subsequent flow experience, which manifests as heightened positive emotional involvement. This AI-supported feedback phase provided a confidence boost not available to the control group, making the experimental group more prone to flow and thus deeper affective engagement.

Higher levels of behavioral engagement were observed among students in the AI–peer integrated feedback group, who showed a stronger tendency to revise their compositions in response to the feedback received. This heightened responsiveness likely attributable to the precision and practical applicability of AI-generated suggestions, which offered explicit, step-by-step guidance for improvement. The structured and instantaneous nature of AI feedback appears to have encouraged learners to undertake more frequent and deliberate revision actions, a finding that aligned with Wang and Xue (2024) observation of positive behavioral effects associated with AI chatbot use. In the present study, behavioral engagement was operationalized through revision operations and revision strategies (e.g., the use of external sources such as online sources). Generally, semi-structured interviews suggested that AI recipients invested more effort in both comprehending and implementing the suggested changes. One plausible explanation is that learners in the peer group bore extra interpretive demands. When processing potentially ambiguous, imprecise peer feedback, learners expended extra effort to interpret suggestions, which might limit their capacity to implement feedback as successfully as their AI-supported counterparts.

With regard to cognitive engagement, students in the control group, who received peer feedback only, operated at a largely superficial level, whereas their counterparts in the experimental group, exposed to a sequential AI-peer feedback integration, exhibited substantially deeper cognitive processing. This finding diverged from Huang and Teng (2025), who reported no significant difference between AI and peer feedback conditions. Such a discrepancy might be attributed to the phased delivery design employed in this study, wherein learners first received AI-generated feedback, revised their drafts accordingly, and subsequently accessed peer feedback for a second round of revision. This phased structure enabled participants to break down the revision task into discrete, manageable stages (Zhang and Hyland, 2022). Interview data corroborated this interpretation, with experimental-group students reporting that they strategically prioritized their revision efforts across phases, addressing lower-order linguistic errors flagged by AI during the initial stage, while reserving higher-order concerns, such as content organization and coherence, for subsequent peer-led discussion. By offloading surface-level corrections to AI, learners were able to free substantial cognitive capacity, allowing them to potentially channel greater attentional resources toward peer feedback targeting global textual dimensions. Consequently, this sequential integration appears to generate additive benefits that surpass those attainable through either feedback source in isolation.

Overall, this study supported Han and Hyland’s (2015) finding, highlighting the interconnectedness of affective, behavioral, and cognitive engagement. Positive affective engagement, underpinned by learner confidence and motivation, might act as a catalyst for deeper behavioral and cognitive engagement. Learners who perceive feedback as supportive devote greater effort to sustained revision and critical reflection, a pattern that was clearly evidenced in the experimental group. Importantly, cognitive and behavioral engagement do not operate in isolation. Rather, they function as mutually reinforcing dimensions that might jointly determine how effectively feedback is processed and translated into L2 writing development.

### Students’ writing performance in both groups

This study compared the effects of AI–peer integrated feedback and peer feedback on Chinese EFL learners’ writing performance. Results showed that AI–peer integrated feedback condition significantly outperformed peer-only feedback across all four dimensions. These quantitative patterns aligned with observations from Zou et al. (2025), who noted that integrating the AI-supported feedback (e.g., feedback from ChatGPT) produced substantial advances in EFL learners’ writing skills and highlighted AI’s potential to elevate overall L2 writing quality. This findings would be attributed to complementary strengths within AI-peer integrated feedback. In this combined framework, AI tools handle surface-level grammatical error detection and resolution, freeing peer reviewers to deliver in-depth feedback targeting content, idea development, and text organization. By contrast, peer-only feedback frequently overemphasizes surface-level grammatical mistakes at the cost of substantive content-oriented comments. Second, AI-driven tools supply immediate, individualized and real-time recommendations for linguistic enhancement (Guo et al., 2024). Unlike peer-only feedback, which often involves delays, AI rapidly identifies specific errors and delivers corrective prompts. This expedited feedback loop might enable more targeted revisions and drives meaningful writing-performance gains for experimental-group participants. Findings might also stem from elevated learner engagement triggered by AI-peer integrated feedback (Zhao, 2025). Due to its immediacy and practical utility, AI-generated feedback fosters low-anxiety, supportive learning conditions. Data from questionnaires, reflective journals, and semi-structured interviews point to a plausible pathway initiated by affective engagement: positive affect first boosts behavioral engagement, prompting learners receiving supportive AI feedback to revise more frequently. Such sustained revision, in turn, might enhance cognitive capacity for higher-order tasks like generating ideas and structuring texts, thus deepening cognitive engagement. Affective engagement therefore might act as a catalyst for enhanced behavioral and cognitive investment (Koltovskaia et al., 2024). Nevertheless, this affective-to-behavioral pathway does not operate in isolation. Behavioral and cognitive engagement reinforce one another reciprocally (Pearson, 2024), and their mutual interplay might govern how thoroughly learners process feedback and translate it into tangible L2-writing improvements.

These results can further be interpreted through sociocultural theory (Vygotsky, 1978), which conceptualizes learning as a socially mediated process shaped by human interaction and cultural tools. Cognitive growth emerges from collaborative activity, where learners draw on external mediation from peers or technological artifacts to expand their capacities. The present study’s AI-peer integrated feedback instantiates this theoretical premise: learners completed peer-feedback activities after receiving real-time ChatGPT suggestions, creating multi-layered mediational support. Dual mediators might scaffold learners’ revision practices, potentially promoting higher-order cognitive engagement, and ultimately might advance L2 writing proficiency. Both the intervention design and empirical outcomes illustrate sociocultural theory’s core claim that peer- or technology-enabled mediation fuels EFL students’ writing development.

Although the experimental group outperformed the control group across all dimensions, the magnitude of improvement varied for students in the experimental group, which aligned with findings reported by Guo et al. (2024). Task achievement and lexical resource yielded substantially larger significant improvements compared with coherence and cohesion, whereas grammatical range and accuracy produced the smallest numerical gains, with no statistically significant differences relative to the other dimensions. This observation might be attributed to AI’s advantages in rhetorical-task analysis. The pronounced improvements in task achievement stem from large-language-model-driven AI systems, which exhibit superior capacity to decompose essay prompts, verify whether all task components have been adequately addressed, and detect argumentative gaps or off-topic elaboration (Yuan et al., 2024). By contrast, peer-only feedback providers often lack sufficient metacognitive awareness of task requirements themselves and tend to generate vague, affectively laden comments (Liu and Sadler, 2003). Lexical resource likewise saw prominent gains. AI systems maintain nearly exhaustive inventories of academic collocations and register-appropriate synonym distinctions, enabling specific, contextualized, and developmentally calibrated feedback (Guo and Wang, 2024). Such feedback makes it easier for learners to retain target synonyms. In contrast, coherence and cohesion yielded more modest improvements, notwithstanding the overall superiority of AI-peer integrated feedback. Coherence and cohesion operates at the supra-sentential and macro-structural discourse level, demanding logical progression across paragraphs, appropriate information sequencing at the essay level, and rhetorical structures consistent with genre conventions (Hyland, 2002). Although AI is capable of identifying missing transition markers or ambiguous referential chains at the sentence level, diagnosing global architectural flaws requires holistic text processing that might exceed the analytical granularity of conventional AI feedback (Stevenson and Phakiti, 2019). Grammatical range and accuracy similarly showed limited improvement. This dimension concerns the use of varied syntactic structures in real-time writing, which depends on procedural rather than declarative knowledge (DeKeyser, 2007). Although AI feedback can identify opportunities for syntactic diversification, converting such declarative awareness into proceduralized production requires sustained practice that a brief intervention cannot provide.

## Conclusion

Using a mixed-methods design, this study investigated the effects of AI-peer integrated feedback on EFL students’ writing engagement and performance. Quantitative and qualitative analyses revealed that students who received AI-peer integrated feedback displayed greater gains in writing performance and demonstrated significantly deeper engagement across behavioral, cognitive and affective dimensions. Qualitative analysis supported these results, revealing that AI-peer integrated feedback potentially contribute to gains in writing quality and engagement through multiple factors: positive attitudes towards AI-produced feedback, active engagement with feedback by implementing revision actions and strategies, greater feedback awareness, and wide application of cognitive and metacognitive strategies.

The outcomes of this study carry substantial practical implications for improving EFL writing pedagogy in Chinese higher-education contexts. To foster Chinese EFL learners’ academic writing quality and engagement levels, learners are encouraged to actively adopt AI resources during revision. Incorporating AI technology offers a feasible approach to deliver more frequent, targeted, and individualized feedback, a pedagogical resource frequently constrained by oversized classrooms and limited teaching hours. Rather than substituting conventional instructional practices, AI-peer integrated feedback acts as a possible instructional resource, allowing peer feedback providers to devote greater attention to higher-order writing components including argument construction and critical reasoning, while AI systems take charge of resolving surface-level linguistic errors.

Beyond classroom-based practice, these findings offer practical suggestions for material developers and curriculum planners. Developers should prioritize writing assignments designed to maximize the strengths of AI-peer integrated feedback. Instructional materials ought to include tasks encouraging learners to critically reflect on AI-generated feedback and engage in iterative revision. Such resources should be user-friendly and flexible to accommodate the broad proficiency spectrum among Chinese university EFL learners. Curriculum planners may embed AI-peer integrated feedback into core writing syllabi alongside peer-feedback activities. This curricular integration must be paired with mandatory training for students and educators to strengthen digital literacy and foster critical evaluation of AI-produced feedback. In the long term, evidence-informed adoption of AI-peer integrated feedback can improve writing performance and learner engagement, while simultaneously fostering Chinese EFL students’ AI literacy and their ability to leverage technological tools in academic contexts.

Several limitations of this study need to be acknowledged. First, the quasi-experimental design (intact-class allocation, single institution) and the gender imbalance within the sample may constrain the generalizability of the findings. Future research would benefit from employing individual-level random assignment, multiple instructors, and more diverse, gender-balanced sampling across institutions to further validate the findings. Second, the absence of a delayed post-test prevents inferences about whether observed writing improvements endure over extended periods. Longitudinal follow-up assessments featuring delayed post-tests are therefore needed to trace the sustainability of writing-related gains. Third, participants’ limited prior exposure to AI-assisted feedback tools raises the possibility that the observed positive outcomes may partly reflect a novelty effect. Longer intervention timelines are therefore needed to distinguish durable intervention effects from transient novelty-driven responses. Lastly, while this study offers preliminary insights into the effects of AI-peer integrated feedback on learners’ engagement and writing, the design precludes definitive causal claims about mediating processes. Future research could employ structural equation modelling with larger samples to test potential mediators, such as feedback literacy, which has recently been found to predict feedback engagement in multilingual contexts (Boudouaia et al., 2026).

## Data Availability

The original contributions presented in the study are included in the article/Supplementary material, further inquiries can be directed to the corresponding author.
