# Supplementary material for: AI-peer integrated feedback in second language writing classes: exploring students’ engagement and writing performance
Source: Front Psychol. 2026 Sep 21;17:1871951. doi: 10.3389/fpsyg.2026.1871951 (PMC13635350; doi:10.3389/fpsyg.2026.1871951)
Supplement: Supplementary file 1 [file Supplementary_file_1.DOCX]

Appendix A.

Pretest

You should spend about 45 minutes on this task.

Write about the following topic:

Some people feel that entertainers (e.g. film stars, pop musicians or sports stars) are paid too much money.
Do you agree or disagree?
Which other types of job should be highly paid?

Give reasons for your answer and include any relevant examples from your own knowledge or experience.

Write at least 200 words.

Posttest

You should spend about 45 minutes on this task.

Write about the following topic:

Being a celebrity – such as a famous film star or sports personality – brings problems as well as benefits.

Do you think that being a celebrity brings more benefits or more problems?

Give reasons for your answer and include any relevant examples from your own knowledge or experience.

Write at least 200 words.

Appendix B. Engagement questionnaire

Instructions: Indicate your level of agreement with each statement on the following 5-point Likert scale: 1 = Strongly disagree; 2 = Disagree; 3 = Neutral; 4 = Agree; 5 = Strongly agree

| Engagement |  |  |  |  |  |
| --- | --- | --- | --- | --- | --- |
| Affective engagement |  |  |  |  |  |
| 1. I enjoy engaging with the feedback I received. | 1 | 2 | 3 | 4 | 5 |
| 2. I feel motivated to revise my writing based on the feedback. | 1 | 2 | 3 | 4 | 5 |
| 3. I am satisfied with the feedback I received. | 1 | 2 | 3 | 4 | 5 |
| 4. I have a negative attitude toward the feedback. | 1 | 2 | 3 | 4 | 5 |
| 5. I find the peer feedback useful and important. | 1 | 2 | 3 | 4 | 5 |
| Cognitive engagement |  |  |  |  |  |
| 1. I can notice the feedback. | 1 | 2 | 3 | 4 | 5 |
| 2. I can clearly understand the meaning and intent of the feedback. | 1 | 2 | 3 | 4 | 5 |
| 3. I critically evaluate the accuracy and usefulness of the feedback. | 1 | 2 | 3 | 4 | 5 |
| 4. Using the feedback, I identify both the strong and weak points in my writing. | 1 | 2 | 3 | 4 | 5 |
| 5. I apply specific strategies to enhance my writing, drawing on the feedback I received. | 1 | 2 | 3 | 4 | 5 |
| 6. I connect the feedback I receive to my writing development. | 1 | 2 | 3 | 4 | 5 |
| Behavioral engagement |  |  |  |  |  |
| 1. I carefully read and review all the feedback. | 1 | 2 | 3 | 4 | 5 |
| 2. I make targeted revisions to my writing based on the feedback. | 1 | 2 | 3 | 4 | 5 |
| 3. I ask clarifying questions when I do not understand the feedback. | 1 | 2 | 3 | 4 | 5 |
| 4. I prioritize addressing the feedback in my writing revisions. | 1 | 2 | 3 | 4 | 5 |
| 5. I complete revisions to my writing in a timely manner after receiving feedback. | 1 | 2 | 3 | 4 | 5 |

Appendix C. Semi-structured interviews for focal students in both group

Instruction for AI-peer integrated feedback group

In this interview, I would like to understand your experiences and perceptions related to the AI-peer integrated feedback you received during the writing course. Please share your thoughts, feelings, and behaviors regarding how you engaged with the feedback throughout the intervention.

Instruction for peer feedback group

In this interview, I would like to understand your experiences and perceptions related to the traditional peer feedback you received during the writing course. Please share your thoughts, feelings, and behaviors regarding how you engaged with the feedback throughout the intervention.

Semi-structured interviews

1. Could you share your past experiences with writing in English?

2. What is your opinion of the current English class?

3. What are your thoughts on the feedback activity?

4. How do you view the feedback you received on your writing?

5. What emotions do you feel when you receive the feedback?

6. In what ways do you apply the feedback to enhance your draft?

7. How do you handle or process the feedback you get?

8. Is the feedback on your writing clear and understandable to you?

9. What methods or approaches do you employ when revising your writing?

10. Do you have any recommendations for improving the feedback activity?

Appendix D. Reflective journals

A reflection prompt adapted from Han (2016) was administered to both groups to guide participating students’ written reflections.

Please write a reflection describing your experiences and feelings while receiving the feedback on your writing over the 12-week semester. You may also note any changes in how well you notice, understand, and process the feedback; how you use the feedback to revise your drafts; and your emotional attitude toward the feedback. You may include any factors that influenced your experience. Your entry should be at 150 words and may be written in Chinese or English.
